# Sarcopenia, Eosinophil-to-Platelet Ratio, and C-reactive Protein as Predictors of Adverse Events in Patients With Acute Exacerbations of Chronic Obstructive Pulmonary Disease: A Prospective Observational Study

**DOI:** 10.7759/cureus.56651

**Published:** 2024-03-21

**Authors:** Rohankumar Gandhi, Vijay Kalsariya, Roshan Katara, Yogesh Murugan

**Affiliations:** 1 Community and Family Medicine, Guru Gobindsingh Government Hospital, Jamnagar, IND; 2 Pulmonary Medicine, Guru Gobindsingh Government Hospital, Jamnagar, IND; 3 Family Medicine, Guru Gobindsingh Government Hospital, Jamnagar, IND

**Keywords:** exacerbation, eosinopenia, c-reactive protein, prognosis, copd

## Abstract

Background: Biomarkers such as sarcopenia, eosinopenia, and C-reactive protein (CRP) may predict adverse events in chronic obstructive pulmonary disease (COPD) exacerbations. We aimed to determine their prognostic utility and accuracy versus conventional measures.

Methods: This was a prospective analysis of COPD patients hospitalized for acute exacerbations for more than one year. Patients with primary diagnoses other than COPD were excluded. A total of 200 participants were screened, and 50 experienced adverse events, including mortality, rehospitalization, prolonged stay, hypoxemia, or hypercapnia. Data on demographics, lung function, symptoms, nutrition, frailty, sarcopenia, the eosinophil-to-platelet ratio (EPR), and CRP were extracted. Differences between groups were analyzed using t-tests and regression modeling.

Results: Elevated CRP and a low EPR were significant predictors of adverse events after adjustment, with CRP having an area under the curve (AUC) of 0.71 (0.64-0.80) and EPR having an AUC of 0.76 (0.61-0.79) for composite outcomes. According to the multivariate logistic regression analysis, sarcopenia (adjusted Or (aOR)-1.97 (1.87-4.44)), EPR (aOR-2.33 (1.02-5.32)), and CRP (aOR-2.09 (1.01-3.18)) remained significant.

Conclusion: The EPR and CRP levels are useful prognostic markers of in-hospital morbidity and mortality during COPD exacerbations. However, multidimensional assessments incorporating other treatable traits may further optimize risk prediction and reduce adverse outcomes.

## Introduction

Chronic obstructive pulmonary disease (COPD) is a major public health problem affecting more than 170 million people globally. It is currently the fourth-leading cause of mortality worldwide, and further increases in its prevalence and mortality are predicted in the coming decades [1]. COPD is characterized by persistent respiratory symptoms and progressive airflow limitation due to abnormalities of the airways and alveoli. The disease course is punctuated by acute exacerbations, which are defined as episodes of worsening symptoms requiring additional treatment. Exacerbations are critical events that are associated with poor health-related quality of life, accelerated lung function decline, increased healthcare utilization, and a greater risk of mortality [2].

Previous studies have identified risk factors for COPD exacerbations, including prior exacerbation history, poor lung function, chronic bronchitis symptoms, bacterial colonization, and low physical activity [3]. However, these known factors do not fully explain the heterogeneity in exacerbation susceptibility between patients. There has been a growing interest in identifying novel predictive biomarkers and treatable traits that may guide more personalized management approaches [4]. In particular, low-grade systemic inflammation and extrapulmonary manifestations are increasingly recognized to contribute to COPD severity. This has led to research on targeted anti-inflammatory therapies and addressing systemic comorbidities to optimize COPD management [5].

One relevant comorbidity is sarcopenia, which is defined as the degenerative loss of skeletal muscle mass and strength. Recent studies have reported that the prevalence of sarcopenia is between 10% and 20% in patients with stable COPD. Sarcopenia likely results from physical inactivity, poor nutrition, systemic inflammation, and other complex mechanisms in COPD patients. Previous studies have linked sarcopenia to impaired exercise capacity, lower quality of life, osteoporosis, and a greater risk of mortality in COPD patients. There is also emerging evidence that sarcopenia is associated with poorer outcomes, including more frequent exacerbations, but data on its predictive utility is limited [6-8].

The peripheral blood eosinophil-to-platelet ratio (EPR) has recently been evaluated as a biomarker of COPD. Eosinophilic airway inflammation is characterized by a distinct COPD phenotype and a good response to corticosteroid therapy [9]. Blood eosinophilia is associated with increased exacerbation risk in COPD patients [10]. The EPR provides a simple and practical way to indicate corticosteroid-responsive disease. However, few studies have evaluated the EPR together with other systemic biomarkers for exacerbation prediction.

C-reactive protein (CRP) is an established marker of systemic inflammation that is elevated in COPD patients. Multiple studies have reported that CRP levels strongly predict COPD exacerbations, hospitalization, and mortality [11]. CRP cut-offs of >3 to 10 mg/L have been proposed to indicate high-risk patients. Whether newer biomarkers such as sarcopenia and the EPR provide additional predictive value over CRP alone is unknown.

In summary, sarcopenia, the EPR, and CRP levels are promising systemic biomarkers that may predict clinically important COPD outcomes, including exacerbations. However, previous studies have evaluated these markers individually, and prospectively validated data are lacking, particularly from non-Western populations. The present study aimed to determine the predictors of adverse events, including mortality, rehospitalization, prolonged hospital stay, hypoxemia, and hypercapnia, in patients hospitalized for acute exacerbation of chronic obstructive pulmonary disease (COPD).

## Materials and methods

This was a prospective observational study conducted at a single tertiary care hospital, Guru Gobind Government Hospital, Jamnagar, in Gujarat, India, over the course of one year (February 2023-February 2024). Written informed consent was obtained from the patients in their vernacular language. Ethical approval was obtained from Shri M P Shah Government Medical College, Jamnagar, Gujarat, India before the start of the study (approval number: 37/01/23).

Sample size estimation

With a power of 80% and an alpha of 0.05, a sample of 180 patients was required to detect a medium effect size of 0.3 for the correlation between predictors and outcomes based on prior studies [11]. After accounting for 10% attrition, the final sample size was 200.

Sampling technique and participant recruitment

Eligible patients were those aged above 40 years with a prior physician diagnosis of COPD based on spirometry and smoking history and hospitalized for acute exacerbation defined by worsening respiratory symptoms from baseline requiring emergency care. Patients were excluded if they had a primary diagnosis other than COPD exacerbation, such as the presence of pneumonia, pneumothorax, pulmonary embolism, or other respiratory conditions, as the primary reason for hospitalization.

Consecutive sampling was used to recruit participants who met the eligibility criteria for hospital admission during the study period. A total of 450 patients admitted with COPD exacerbations were screened, of whom 206 met the eligibility criteria and were enrolled in the study after providing informed consent. Of these 206, six were subsequently excluded due to a lack of follow-up. The remaining 200 participants were followed throughout their hospitalization to ascertain the occurrence of predefined adverse events, including mortality, rehospitalization, prolonged stays of more than five days, hypoxemia, and hypercapnia. All participants received standard medical care, which included bronchodilators, steroids, antibiotics, oxygen therapy, and other interventions as clinically indicated, as determined by the treating physicians. These included respiratory support (non-invasive ventilation, mechanical ventilation), fluid management (intravenous fluids, diuretics), treatment of comorbidities (e.g. heart failure management, anticoagulation), and pulmonary rehabilitation.

Baseline data were collected for all participants using standardized techniques and operational definitions. Various clinical and laboratory parameters were collected through a review of medical records, including anthropometric measurements, spirometry results, serum CRP levels, nutritional status, COPD assessment test (CAT) scores, and frailty assessments. The baseline characteristic data were compared between the adverse event and nonadverse event groups for further analysis.

In this study, among the 200 participants included in the final analysis, 50 experienced one or more adverse events during their hospital stay. Thus, 50 patients in the adverse event group and 150 in the nonadverse event group were enrolled and evaluated.

Data collection

A standardized data collection form was used to extract information from electronic medical records on demographics, smoking status, anthropometric data, laboratory investigations, length of stay, complications, and mortality. Data on predictor variables, including sarcopenia (assessed using bioimpedance analysis to estimate muscle mass, combined with grip strength for muscle function, per the European Working Group definition), EPR (a calculated ratio using the eosinophil and platelet counts from a complete blood count laboratory test), CRP (a blood test measuring levels of this inflammatory biomarker.), COPD assessment test (a validated patient-reported questionnaire scoring symptoms and impacts of COPD), mini nutritional assessment-short form (MNA-SF) (a standardized questionnaire and simple measurements to screen for malnutrition risk), and frailty index were obtained. The outcomes collected were all-cause mortality, hospital readmission within 30 days of discharge, hospital stay longer than five days, and arterial blood gas evidence of hypoxemia or hypercapnia during admission.

Variables and operational definitions

The independent variables are as follows: elevated CRP was defined as CRP ≥15.8 mg/dL [11], CAT score measured at admission >20 indicated the impact of COPD on health status [12], sarcopenia was defined as low muscle mass and function according to the European Working Group criteria-2 using bioimpedance analysis [13], low EPR was <0.755 [14], MNA-SF score range was 0-14; <12 indicated malnutrition risk [15], frailty was assessed using the Clinical Frailty Scale 1-9 and a score ≥5 indicated frailty [16].

The dependent variables are as follows: Mortality indicated all-cause mortality during hospitalization, Rehospitalization indicated COPD readmission within 30 days of discharge, Prolonged stay indicated a length of stay >5 days, Hypoxemia indicated partial pressure of oxygen (PaO2) <60 mmHg on arterial blood gas, and hypercapnia indicated PaCO2 >50 mmHg [17].

Data analysis

Descriptive statistics, including means, standard deviations, and percentages, were used to characterize the sample. Bivariate comparisons between groups were performed using independent t-tests, Mann‒Whitney U tests, and chi-square tests, as appropriate. Correlations were assessed using Pearson's correlation coefficient. Logistic regression was used to identify independent predictors of adverse events. Diagnostic accuracy was evaluated using sensitivity, specificity, and area under the receiver operating characteristic curve (AUC). The data were analyzed using IBM SPSS Statistics for Windows, Version 25.0 (Released 2017; IBM Corp., Armonk, New York, United States), and a p-value <0.05 was considered to indicate statistical significance.

## Results

Table 1 shows the baseline characteristics of COPD patients with and without adverse events. Patients with adverse events had significantly lower BMIs (21.1 vs 22.5, p=0.04), higher CAT scores (25.1 vs 22.3, p=0.01), lower MNA-SF scores (10.2 vs 11.8, p<0.001), higher rates of frailty (36% vs 21.3%, p=0.04), lower EPRs (70% vs 44.7% with EPR <0.755, p=0.002), higher rates of sarcopenia (50% vs 30%, p=0.01), and higher CRP levels (12.5 vs 7.8, p<0.001).

**Table 1:** Baseline characteristics of COPD patients with and without adverse events during hospitalization for acute exacerbation P<0.05 *-significant, p<0.001-highly significant. FEV1: forced expiratory volume in the first second; CAT: chronic obstructive pulmonary disease assessment test; MNA-SF: Mini Nutritional Assessment-Short Form; EPR: eosinophil-to-platelet ratio; CRP: C-reactive protein

Characteristic	Adverse Events (n = 50)	No Adverse Events (n = 150)	P value
Age (years), mean±SD	67.5 ± 8.2	65.8 ± 7.9	0.23
Male sex, n (%)	32 (64%)	94 (62.7%)	0.88
Smoking (pack-years), mean±SD	46.3 ± 25.7	42.1 ± 21.2	0.21
BMI (kg/m^2^), mean±SD	21.1 ± 3.8	22.5 ± 4.1	0.04 *
FEV1% predicted (%), mean±SD	45.2 ± 15.1	48.7 ± 13.5	0.18
CAT score, mean±SD	25.1 ± 6.3	22.3 ± 5.8	0.01 *
MNA-SF score, mean±SD	10.2 ± 2.1	11.8 ± 1.9	<0.001 **
Frailty, n (%)	18 (36%)	32 (21.3%)	0.04 *
EPR <0.755, n (%)	35 (70%)	67 (44.7%)	0.002 *
Sarcopenia, n (%)	25 (50%)	45 (30%)	0.01 *
CRP (mg/L), mean±SD	12.5 ± 8.1	7.8 ± 5.2	<0.001 **

Table 2 shows the correlations between the predictors. Strong correlations were detected between sarcopenia and the EPR (r=-0.42, p<0.001), between sarcopenia and CRP (r=0.31, p=0.002), between the EPR and CRP (r=-0.39, p<0.001), between frailty and the MNA-SF score (r=-0.57, p<0.001), and between the CAT score and CRP (r=0.29, p=0.004).

**Table 2:** Correlations between predictors P<0.05 *-significant, p<0.001-highly significant. CAT: chronic obstructive pulmonary disease assessment test; MNA-SF: Mini Nutritional Assessment-Short Form; EPR: eosinophil-to-platelet ratio; CRP: C-reactive protein

Predictor 1	Predictor 2	Correlation coefficient	P value
Sarcopenia	EPR	-0.42	<0.001 **
Sarcopenia	CRP	0.31	0.002 *
EPR	CRP	-0.39	<0.001 **
Frailty	MNA-SF score	-0.57	<0.001 **
CAT score	CRP	0.29	0.004 *

Table 3 displays the bivariate associations between predictors and adverse events. According to the unadjusted analysis, sarcopenia (crude OR (cOR) 2.33, p=0.02), EPR<0.755 (cOR 3.04, p=0.003), CRP≥15.8 mg/dL (cOR 1.15, p=0.001), frailty (cOR 2.14, p=0.047), MNA-SF score (cOR 0.76, p=0.001), and CAT score (cOR 1.09, p=0.01) were associated with adverse events. According to the adjusted analysis, sarcopenia (adjusted OR (aOR) -1.97 (1.87-4.44)), EPR (aOR-2.33 (1.02-5.32)), and CRP (aOR-2.09 (1.01-3.18)) remained significant.

**Table 3:** Associations between predictors and adverse events (Multivariate logistic regression) P<0.05 *-significant, p<0.001-highly significant. CAT: chronic obstructive pulmonary disease assessment test; MNA-SF: Mini Nutritional Assessment-Short Form; EPR: eosinophil-to-platelet ratio; CRP: C-reactive protein

Predictor	Crude OR (95% CI)	Adjusted OR (95% CI)	P value
Sarcopenia	2.33 (1.12-4.85)	1.97 (1.87-4.44)	0.02 *
EPR <0.755	3.04 (1.47-6.28)	2.33 (1.02-5.32)	0.003 *
CRP ≥15.8 mg/dL	1.15 (1.06-1.25)	2.09 (1.01-3.18)	<0.001 **
Frailty	2.14 (1.01-4.53)	1.67 (0.71-3.93)	0.24
MNA-SF score	0.76 (0.65-0.89)	0.85 (0.71-1.01)	0.06
CAT score	1.09 (1.02-1.17)	1.05 (0.98-1.13)	0.16

Table 4 shows the diagnostic performance for specific adverse events. For mortality, an EPR <0.755 had the best AUC of 0.72. For hospital readmission, CRP ≥15.8 mg/dL had the highest AUC of 0.69. For prolonged stays, CRP again had the best AUC of 0.73. For hypoxemia, the EPR had the highest AUC of 0.67, while for hypercapnia, the EPR again had the best AUC of 0.66.

**Table 4:** Diagnostic performance tables for each specific adverse event EPR: eosinophil-to-platelet ratio; CRP: C-reactive protein; AUC: area under the curve

Test	Outcome	Sensitivity	Specificity	AUC (95% CI)
Sarcopenia	Mortality	63%	62%	0.67 (0.58-0.76)
EPR <0.755		71%	59%	0.72 (0.64-0.80)
CRP ≥15.8 mg/dL		79%	53%	0.73 (0.65-0.81)
Sarcopenia	Repeated Hospitalization	46%	70%	0.61 (0.52-0.70)
EPR <0.755		51%	75%	0.68 (0.59-0.77)
CRP ≥15.8 mg/dL		62%	67%	0.69 (0.60-0.78)
Sarcopenia	Prolonged Hospital Stay	55%	71%	0.67 (0.58-0.76)
EPR <0.755		61%	74%	0.72 (0.63-0.81)
CRP ≥15.8 mg/dL		71%	62%	0.73 (0.64-0.82)
Sarcopenia	PaO2 <60 mmHg	41%	71%	0.59 (0.50-0.68)
EPR <0.755		48%	79%	0.67 (0.58-0.76)
CRP ≥15.8 mg/dL		58%	67%	0.66 (0.57-0.75)
Sarcopenia	PaCO2 >50 mmHg	44%	69%	0.60 (0.51-0.69)
EPR <0.755		51%	73%	0.66 (0.57-0.75)
CRP ≥15.8 mg/dL		62%	64%	0.68 (0.59-0.77)

Table 5 displays the overall diagnostic accuracy. An EPR coefficient <0.755 had the best combination of sensitivity (68%) and specificity (77%) for detecting adverse events, with an AUC of 0.76. CRP had a sensitivity of 58%, a specificity of 63%, and an AUC of 0.71. The other predictors performed worse.

**Table 5:** Overall diagnostic performance of the predictors of adverse events CAT: chronic obstructive pulmonary disease assessment test; MNA-SF: Mini Nutritional Assessment-Short Form; EPR: eosinophil-to-platelet ratio; CRP: C-reactive protein; AUC: area under the curve

Test	Sensitivity	Specificity	AUC (95% CI)
Sarcopenia	50%	70%	0.62 (0.53-0.71)
EPR <0.755	68%	77%	0.76 (0.61-0.79)
CRP ≥15.8 mg/dL	58%	63%	0.71 (0.64-0.80)
Frailty	36%	79%	0.59 (0.50-0.68)
MNA-SF score <12	62%	69%	0.68 (0.59-0.77)
CAT score >20	51%	71%	0.64 (0.55-0.73)

**Figure 1:**
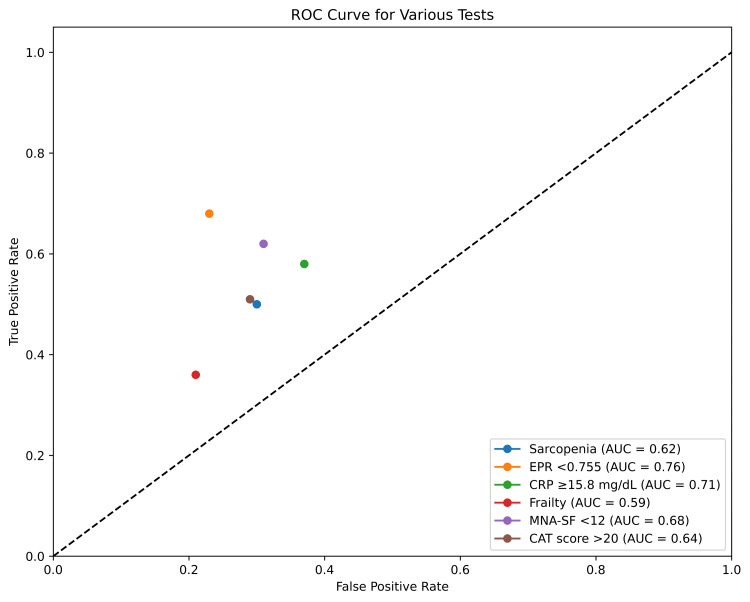
Overall diagnostic accuracy of the predictors CAT: chronic obstructive pulmonary disease assessment test; MNA-SF: Mini Nutritional Assessment-Short Form; EPR: eosinophil-to-platelet ratio; CRP: C-reactive protein; ROC: receiver operating characteristic; AUC: area under the curve

Figure 1 shows the receiver operating characteristic curve of the overall diagnostic performance of the predictors.

## Discussion

This study revealed several significant predictors of adverse events in COPD patients hospitalized for acute exacerbations. A lower EPR and a higher CRP were the strongest predictors, consistent with prior research [11,13,14]. However, other factors, such as frailty, poor nutrition, and symptom severity, also demonstrated predictive value in the bivariate analysis.

The prevalence of sarcopenia was significantly greater in patients who experienced adverse events. Skeletal muscle depletion is known to be associated with mortality, readmission, and length of stay in COPD patients [18]. Possible mechanisms are related to impaired respiration, decreased immunity, and reduced functional capacity [19]. Our study further highlights the importance of assessing muscle mass and quality in hospitalized COPD patients.

Frailty was also more common in patients with adverse events, similar to recent studies [20]. Frailty indicates multisystem dysregulation and poor resilience to stressors. The strong correlation between frailty and nutritional status found here emphasizes the value of a multidimensional prognostic approach in COPD patients.

Poor nutritional scores on the MNA-SF predict complications, reflecting malnutrition's contribution to poor COPD outcomes [21]. Systemic inflammation can suppress appetite and drive hypermetabolism in COPD patients [22]. Ensuring adequate caloric and protein intake during hospitalization through nutritional interventions could help reduce adverse events.

Higher COPD symptom scores in the CAT lost their predictive significance for adverse events after adjusting for other variables in the multivariate analysis. In contrast, more objective physiological measures like sarcopenia, EPR, and CRP retained their predictive value. This suggests that while patient-reported symptom scores like the CAT can provide an initial assessment, they may not fully capture the physiological derangements and systemic manifestations that contribute to adverse outcomes in COPD exacerbations. The key reason is that COPD is a multi-component disease with pulmonary and systemic manifestations. Patient-reported scales alone may not fully predict outcomes. Incorporating comprehensive physiological testing can better characterize the overall disease burden and severity to risk-stratify COPD patients during exacerbations.

Some limitations should be noted. As a single-center analysis, the results may not be generalizable to other settings. We lacked follow-up data on outpatient outcomes. Our sample size provided adequate power for primary analyses but limited subgroup comparisons. Residual confounding is possible, although we adjusted for key covariates. The lack of information on nutritional intervention and counseling is another potential limitation of the study. We suggest that future studies should evaluate the effects of nutritional supplementation/support specifically in sarcopenic COPD patients during exacerbations.

The consistent predictive value of the EPR and CRP across endpoints further establishes their utility for risk stratification and suggests that targeted treatment approaches warrant investigation in this high-risk population. However, sarcopenia, frailty, malnutrition, and symptoms can also identify patients requiring greater support. A multimodal prognosis approach incorporating clinical and laboratory findings may optimize predictions and lead to improved exacerbation outcomes.

## Conclusions

In the present study, the EPR and CRP levels were found to be useful objective predictors of in-hospital morbidity and mortality among COPD patients who experienced acute exacerbations. Screening for these biomarkers on admission could help identify high-risk patients who need more aggressive monitoring and treatment. Additional prospective studies are warranted to validate these findings and inform interventional trials targeting these biomarkers.
